# Effect of glenohumeral forward flexion on upper limb myoelectric activity during simulated mills manipulation; relations to peripheral nerve biomechanics

**DOI:** 10.1186/1471-2474-15-288

**Published:** 2014-09-02

**Authors:** Marinko Rade, Michael Shacklock, Saara M Rissanen, Stanislav Peharec, Petar Bačić, Corrado Candian, Markku Kankaanpää, Olavi Airaksinen

**Affiliations:** Department of Physical and Rehabilitation Medicine, Kuopio University Hospital, P.O. Box 1607, 70211 Kuopio, Finland; “Prim. dr.Martin Horvat” Orthopaedic and Rehabilitation Hospital, Luigi Monti street n.2, 52210 Rovinj, Croatia; Neurodynamic Solutions, Adelaide, Australia; Department of Applied Physics, University of Eastern Finland, Kuopio, Finland; Polyclinic for Physical and Rehabilitation Medicine Peharec, Pula, Croatia; Regional Institute for the Studies on Social Services (I.R.S.Se.S), Trieste, Italy; Department of Physical and Rehabilitation Medicine, Tampere University Hospital, Tampere, Finland

**Keywords:** Tennis elbow, Radial nerve, Mills manipulation, Nociceptive flexion reflex, Neurodynamics

## Abstract

**Background:**

It is generally accepted that muscles may activate via the common nociceptive flexion reflex (NFR) in response to painful stimuli associated with tensile or compressive forces on peripheral nerves. Following the basic assumption that the radial nerve may be stressed around the elbow during the execution of the Mills manipulation, *t* wo positions considered to have different mechanical effects on the radial nerve and the brachial plexus were tested in order to i) explore whether muscles are activated in certain patterns with concomitant changes in nerve tension, ii) establish whether muscle responses can be modified with mechanical unloading of the brachial plexus.

**Methods:**

Muscle responses were quantified bilaterally in eight subjects (N = 16) during Mills Manipulation (MM) pre-manipulative positioning and a Varied position that putatively produces less mechanical tension in the brachial plexus. End range pre-manipulative stretch was used in order to simulate the effects of Mills manipulation. Electromyographic signals were recorded with a 16 channel portable EMG unit and correlated with kinematic data from three charge-coupled device adjustable cameras which allowed for precise movement tracking.

**Results:**

Compared with the Standard Mills manipulation position, the Varied position produced significantly reduced myoelectric activity (*P* **≤** .001) in all test muscles. Additional subjective data support the notion that certain muscle activity patterns were protective.

**Conclusion:**

It seems that protective muscles are selectively activated in a specific pattern in order to protect the radial nerve from mechanical tension by shortening its pathway, suggesting integration of muscle and neural mechanisms. Furthermore, the significantly decreased myoelectric activity with reduced mechanical tension in the brachial plexus may help controlling collateral effects of the Mills manipulation itself, making it potentially safer and more specific.

**Electronic supplementary material:**

The online version of this article (doi:10.1186/1471-2474-15-288) contains supplementary material, which is available to authorized users.

## Background

In the clinical diagnosis and treatment of disorders that affect neural structures in the body, for instance carpal and supinator tunnel and radiculopathy syndromes, different clinically applied physical tests have been created. On a mechanisms level, such manoeuvres move, apply force to, and test the responsiveness of, the relevant nerve structure so the clinician can obtain an impression of its state and mechanical function. These physical tests are currently termed ‘neurodynamic’ tests [1], of which there is a number of manoeuvres that relate to different peripheral nerves of the upper limbs, namely the median, ulnar and radial [2–6]. Since this study focuses on normative responses which may in the future serve as reference material for the Mills manipulation in patients with lateral elbow pain, the manoeuvre relevant to this study is the radial neurodynamic test (RNT). Using buckle force transducers in cadavers, this test has been shown to apply a significant magnitude of tensile force to the radial nerve, as well as to the medial, posterior and lateral cords of the brachial plexus [7]. The same test has also been shown to produce symptoms its end range in asymptomatic subjects [8].

The RNT involves passive scapular depression, shoulder girdle abduction and internal rotation, elbow extension, forearm pronation and wrist and finger flexion to the point of symptom production [2, 5, 6].

Tennis elbow, lateral epicondylitis and lateral epicondylalgia are the terms commonly applied to a condition affecting the myotendinous common extensor origin (CEO) as it inserts into the lateral epicondyle of the humerus, leading to pain and loss of function of the affected limb.

The Mills manipulation was first described by the English orthopaedic surgeon Sir G.Percival Mills [9] from the initial observation that, in patients with tennis elbow, the elbow joint could not be fully extended when this movement was combined with full forearm pronation and wrist and finger flexion. Notwithstanding various theories concerning the underlying therapeutic mechanisms [10–13], the manoeuvre remains current in manual and musculoskeletal medicine practice for the treatment of the lateral elbow pain [14].

As described by Kesson and Atkins [13] and Atkins, Kerr and Goodlad [10], the clinician stands behind the patient and, supporting the patient’s elbow, takes the patient’s shoulder to approximately 90° abduction and allows the shoulder girdle joint to passively settle into an appropriate amount of internal rotation. The clinician then fully flexes the patient’s wrist and completes forearm pronation and elbow extension. At this point, the patient’s elbow reaches the position in which a high velocity-low amplitude thrust toward elbow extension should be applied, and that we, in this paper, define as the ‘pre-manipulative stretch position for Mills manipulation’.

**Figure 1:**
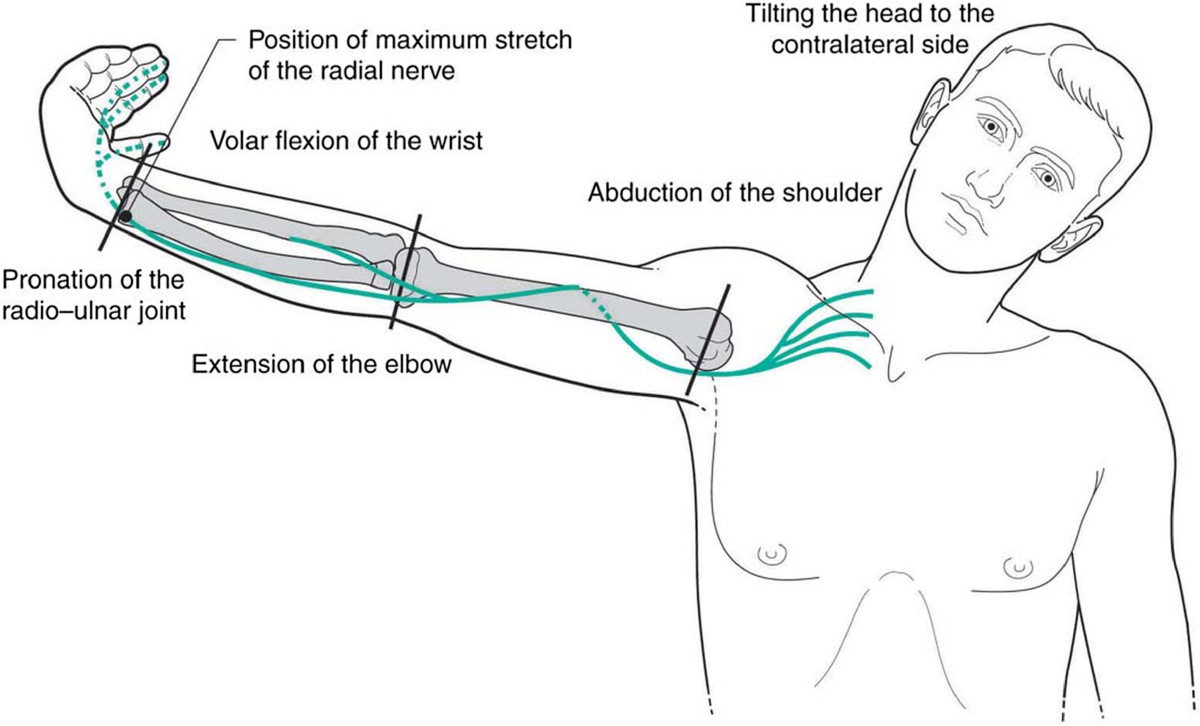
**RNT as first represented by Von Lanz and Wachsmuth**[6]. Note the similarity between Mills manipulation pre-manipulative stretch and this maneuver. From: Shacklock [5], Clinical Neurodynamics, Elsevier, with permission, after Von Lanz and Wachsmuth [6], Praktische Anatomie, Springer Verlag, Berlin. (iii) Radial neurodynamic test (von Lanz T and Wachsmuth W 1959 Praktische Anatomie, Springer-Verlag, Berlin, p. 47, with permission).

Even if this technique were originally designed to stretch the CEO [9, 10, 13, 14] it is pertinent that the final position in which the upper limb is positioned before applying the elbow extension thrust is similar to the one reached during the RNT which is specifically designed to apply mechanical tension to the radial nerve and its posterior interosseous branch. Moreover, shoulder abduction and elbow extension increase mechanical tension in the radial and contiguous nerves, the effects of which are cumulative between these two component movements [15]. Furthermore, it seems that the critical factor in increasing strain in the nerves around the humerus is shoulder abduction to 90° [16] (Figure 1).

Furthermore, even though treatment with Mills manipulation is designed to reduce pain, in practice, this final movement is often described by patients as painful and uncomfortable.

In investigating possible non-specific neural and muscular effects of Mills manipulation, a study by Rade et al. [17] utilized a pre-manipulative stretch for Mills manipulation to measure myoelectric activity in response to changing neural tension in the limb. It was shown that myoelectric activity in certain muscles occurred with this pre-manipulative stretch for Mills manipulation and this activity changed with performance of ipsilateral lateral flexion of the cervical spine; a movement that produces a reduction in tension in the brachial plexus and its more distal nerves. Specifically, the muscles whose activity decreased were those that protect the elbow joint from the movements of the Mills manipulation, elbow extension and forearm pronation. The conclusion was that protective muscle actions at the elbow were likely influenced by neural tension.

In the present paper the investigators are exploring two points: i) whether any discernible pattern in electromyographic activity would emerge during the execution of the pre-manipulative stretch for Mills manipulation in selected muscles, possibly reflexively activated in order to protect the peripheral nerves from excessive mechanical forces in the most logical way; by shortening their pathway and ii) whether non-specific neural and muscular effects of Mills manipulation could be controlled with forward flexion of the shoulder girdle joint; with “non-specific” meaning “collateral effects on adjacent neural structures that may need controlling during the clinical procedure”. In this context, to reduce the neural aspect would be to make the manipulation more localized to the target with fewer non-specific effects.

Forward flexion of the glenohumeral joint was chosen because the brachial plexus passes anterior to the shoulder joint and posteriorly angulated shoulder movements are likely to apply tension to the plexus through a pulley effect on the plexus as it passes in front of the joint. The corollary is that anteriorly angulated movements would do the opposite, thus decreasing tension in the plexus. More specifically, shoulder girdle forward flexion would decrease the mechanical forces applied to the brachial plexus by i) decreasing the distance between the lower cervical region and the axilla, ii) increasing the area of the thoracic outlet tunnel as the clavicle moves anteriorly away from the first and second ribs, while not modifying any other component of the Mills manipulation manoeuvre.

In the present study the authors therefore tested the hypothesis that, in the pre-manipulative stretch position for Mills manipulation, recorded electromyographic (EMG) activity in selected muscles would reduce with reduction in mechanical tension in brachial plexus induced by 65° forward flexion of the shoulder girdle joint compared with the standard position that is utilized clinically (neutral flexion/extension). As such, the pre-manipulative stretch for Mills manipulation was performed in two positions, the Standard (neutral shoulder girdle flexion/extension) and Varied position (shoulder girdle forward flexion to 65°).

The results in this study will provide the basis for clinical comparisons with such normative data.

## Methods

### Subjects

Eleven healthy university student volunteers were recruited and screened for inclusion and exclusion criteria (see Table 1). Three subjects (2 males and 1 female) satisfied the exclusion criteria and were eliminated.

**Table 1:** **Exclusion and inclusion criteria**

Exclusion Criteria:	
• Subjects currently experiencing pain or symptoms on the tested side	• Previous surgery on tested side, including the cervical spine, shoulder, elbow, wrist and hand
• Subjects who did not have pain-free-full range of movement of the cervical spine or shoulder, elbow, wrist and hand joints bilaterally	• Any known structural abnormality of the upper limb
• Previous history of lateral elbow symptoms	• Associated diagnosed neck disorders, lesions including those of the cervical spine and brachial plexus
• History of known neurological disorders of the tested extremity	• More than 3 corticosteroid injections in the last 3 months
• Other joint involvement, like arthritis or already recognized metabolic bone disease	• Associated shoulder disorders, previous trauma with fracture
• Associated wrist disorders or previous trauma of the cervical spine, shoulders, elbows or wrist, bilaterally.	• Subjects older than 55 years to reduce likelihood of significant degenerative changes.
• Subjects with any known arthrogenic, muscular or neurogenic dysfunctions in the cervical spine area which, on provocative physical testing, gave positive signs and/or pain into the arm.	
**Summary of exclusion criteria:** *All the volunteers were screened to be fully asymptomatic and to have a pain-free and complete range of bilateral movement in the cervical spine, shoulders, elbows and wrist and did not match the exclusion criteria.*	
**Inclusion Criteria:**	
• Subjects assessed to be asymptomatic	
• Subjects’ consent to participation by signing the consent form	
• No present exclusion criteria at the time of testing	

Eight remaining volunteers (7 male, 1 female), mean age 28.62 (SD 2.28), BMI mean 24.68 (SD 3.16), all right-handed, were tested bilaterally, providing a sample of 16 recordings.

All tested subjects signed an informed consent form and the study was approved by the Middlesex University, School of Health and Social Sciences Ethics Sub-committee, (London, UK), reference number 595. The study was performed in accordance with the Declaration of Helsinki (1975).

### Electromyographic recordings

Electromyographic signals were recorded with a 16 channel pocket EMG patient unit (POCKETEMG, BTS spa, Garbagnate Milanese, Milano, Italy). Data were collected at a rate of 1000 Hz using a 16-bit A/D board. Electromyographic signals were band passed with a Butterworth filter at 10–499 Hz with a common mode rejection ratio of >100 dB at 65 Hz, an input impedance of >10 GΩ, and transmitted via Wi-Fi to a computer so that the data could be processed off-line using the BTS smart analyzer program.

The EMG signals were smoothed with a root mean square function with a 50 msec sliding window and the area under the processed signals was integrated within five seconds, during which the pre-manipulative stretch was maintained in order to provide quantitative measures of the amount of muscular activation. A 50 msec sliding window was selected following the rationale that the authors were investigating gradual muscular reactions to slow passive movements, rather than performing on-off analysis. The results of the analysis were then exported to a Microsoft Excel program so that the data could be statistically analyzed and displayed graphically.

As the investigators were dealing with myoelectric signals of low amplitude, appropriate steps were taken in the data collection process to exclude background and power line noise from the recorded signals. A laptop working on battery was used and the cables connecting the electrodes to the EMG device were twisted as to diminish the loops.

Also, the power spectrum of each individual signal was checked for 50 Hz peaks using the Discrete Fourier Transformation function included in the analysis tool provided by the manufacturer before accepting the signals for further analysis.

Moreover, the records were double-checked by a second laboratory (Biosignal Analysis and Medical Imaging Laboratory at University of Eastern Finland, Kuopio, Finland) by calculating the Fourier-based spectrum with the Welch’s averaged periodogram method (length of overlapping epochs was 1000 ms and overlap was 75%). No peaks were observed in the spectra in the 50 Hz frequency bin, allowing the investigators to refuse the existence of background noise and power line interference in the recorded signals.

Disposable disc surface foam Ag/AgCl electrodes for EMG recording (Ambu®, Ballerup, Denmark, model N-00-S Blue sensor) were secured over the selected muscles.

Electrode application and skin preparation followed the recommendation of the European Society of Surface Electromyography [18]. The skin was shaved and lightly abraded until the appearance of a light red color and cleaned with distilled water prior to electrode placement. Skin impendence was measured at every electrode placing site with a dedicated device (EMG electrode impedance tester–Noraxon inc, USA) and was verified to be less than 5 kOhm (kΩ) in all electrode sites. Each electrode pair was positioned in bipolar configuration and was placed near the center of the muscle bellies, parallel to the direction of muscle fibers with a fix inter-electrode distance measured between the centers of the electrodes of 30 mm in order to gather information from a sufficient number of motor units, as in Hashimoto et al. [19], Rissanen et al. [20] and Rissanen et al. [21].

The common ground electrode was placed over the spinous process of the C7 vertebrae.

Maximal voluntary contraction (MVC) data normalization was avoided for three reasons. First, a maximal voluntary contraction of the tested muscles would not be suitable for the normalization of the EMG activity recorded during a passive movement. Second, it was possible that the electrical response of muscle during passive nerve stretch could be influenced by muscular fatigue due to the prior contraction. Third, it was decided that the EMG data should be presented as absolute rather than normalized values because authors were comparing relative changes in EMG activity between the two positions (Standard and Varied positions). Compared values were the integral of the EMG signal in 5 second time period in which the pre-manipulative stretch was maintained. This procedure is consistent with those reported in Jaberzadeh et al. [22].

### Kinematics

**Figure 2:**
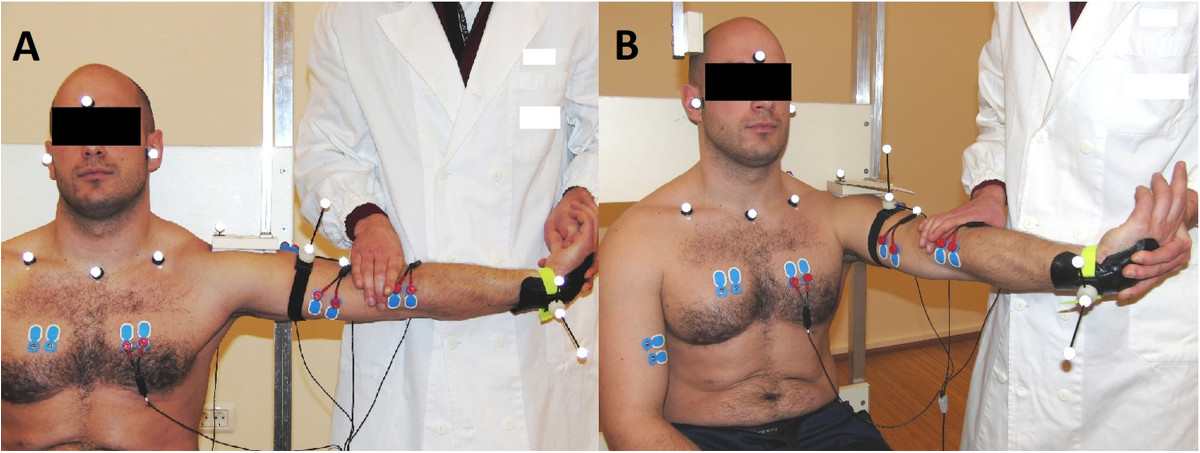
**Pre-manipulative stretch for Mills manipulation performed in the two tested positions. A**. left, standard position, **B**. right, shoulder forward flexion by 65° on the transverse plane. Positions of 10 mm reflective markers and EMG electrodes for a left test side are also visible. Photography release form signed by the volunteer.

The kinematic data were collected with an optoelectronic motion capture system (Smart-D, BTS, Garbagnate Milanese, Milano, Italy) with a 3 charge-coupled device (CCD) cameras adjustable system (sampling frequency of 50 Hz). This was done to correlate joint passive movements with changes in EMG activity. As shoulder abduction and wrist flexion were kept stable by external fixators (Figure 2), particular interest was in verifying full elbow extension, as this may affect the tension of the nerves passing on the anterior side of the elbow joint.

Ten millimeter reflective passive markers were applied on the skin at specific locations, in accordance with the International Society of Biomechanics recommendations on joint coordinate systems in upper limbs [23]. Such locations consisted of the temporomandibular region (bilaterally), mid-frontal region, interclavicular notch, mid-clavicle (bilaterally).

The temporomandibular and mid-frontal region markers were inserted in order to ascertain that no ipsilateral lateral cervical flexion during the maneuvers performance occurred, as it has already been shown in a previous investigation [17] to decrease the myoelectric activity in potentially protective muscles (biceps brachii and brachioradialis) for nerves passing in front of the elbow joint.

Furthermore, the range of motion of elbow extension was measured during pre-manipulative stretch in both the Standard and the Varied (forward flexion) positions with a full-circle hand goniometer with one-degree increments following the directions of the American Academy of Orthopedic Surgeons [24] and verified off-line with the optoelectronic tracking system mentioned above.

### Tested muscles

The choice of test muscles was based on their capacity to produce protective effects by: i) shortening the pathway of the peripheral nerves passing on the concave (anterior) side of the elbow joint (biceps brachii and brachioradialis), ii) shortening the neural pathway in the shoulder region by shortening the distance between the axilla and the lower cervical spine (upper trapezius and pectoralis major), or because they were directly innervated by the radial nerve (brachioradialis and lateral head of the triceps brachii) [25, 26].

In contrast, the lower trapezius muscle was used as a “non-protective muscle” to verify that changes in EMG activity in the other muscles were not simply due to a non-specific increase in muscle activity of the tested upper limb in response to the pre-manipulative stretch. The lower trapezius is connected to the test limb by its scapular insertion, but is not directly innervated by the radial nerve and it is here thought not to participate in the protection mechanism supposedly operating by shortening the neural pathway, making his activation discordant with those of the test muscles. For these reasons it is considered to be a suitable comparative muscle.

### Test manoeuvers: pre-manipulative stretch for mills manipulation

Subjects were seated and, as with other investigations into neurodynamic tests, overall consistency in posture was achieved by means of a custom-made device designed to prevent scapular elevation and cervical movements [27–29].

Support bracing was also applied at the wrist for consistency in joint posture. The wrist was therefore maintained at an angle of 70° flexion, in accordance with Kleinrensink et al. [7].

The pre-manipulative stretch itself consisted of a passive maneuver in which the investigator would hold the subjects’ elbow firmly at the end-range position for Mills manipulation in shoulder girdle joint abduction to 90° and medial rotation, elbow extension, forearm full pronation, with wrist and finger flexion for five seconds.

In order to establish if an effect on muscle activity occurred with shoulder girdle positioning, the pre-manipulative stretch was performed in two different postures,

A. Standard position, the usual position as applied clinically as in Atkins, Kerr et al. and Kesson and Atkins [10, 13], and B. Varied position involving 65° forward flexion of the shoulder while maintaining the 90° elevation in frontal plane (Figure 2).

As opposed to performing the sudden manipulative thrust, the pre-manipulative stretch technique was chosen because; A. the rapid movement of a manipulation was likely to produce significant movement-related electromyographic artifacts that would contaminate the EMG data, B. the small amplitude high-velocity thrust was likely to amplify the myoelectric data already recorded during the pre-manipulative positioning due to an increased instantaneous tension in the affected tissues which would not add useful information and, C. the manipulative thrust would raise ethical concerns because the manoeuvre is usually painful and may produce unwanted structural changes in healthy subjects.

The two different postures were performed in random order on every subject in order to prevent any test bias and avoid the possibility that a recorded abnormal or anomalous electromyographic pattern could partly be a consequence of the order of performance.

The tested subjects were fully blinded to basic differences between the manoeuvres that were performed, and no useful information could be extrapolated from the performance succession pattern.

### Subjective data collection

A simple questionnaire was given to all the tested subjects immediately after testing, where it was asked which of the two manoeuvres was felt to be the more or less painful. The term ‘painful manoeuvre’ was associated with the position less likely to be held before a short amplitude-high velocity elbow extension thrust.

### Statistical analysis

The purpose of the data analysis was to detect any statistically significant differences in the amplitude values of electrical responses in the test muscles when the pre-manipulative stretch was performed in the two different positions, Standard and Varied, using the EMG data recorded in the two tested positions. The hypothesis that electrical responses in the Varied position were less than in the Standard position versus no change was tested. As the conditions for application of parametric tests were not satisfied, Wilcoxon matched-pairs signed-ranks non-parametric testing was used, since this method can be applied to small samples. The alpha level was set at *P =* .05.

The Observed Power was calculated on the data using Wilcoxon matched-pairs signed test, while the minimum number of subjects needed to extract statistically significant results was calculated from the collected data.

The Effect size was calculated dividing the mean value by the Cohen’s *d*_*z*_ value, as suggested in Lakens [30] while 95% Confidence Intervals (CI) were calculated using t-distribution.

Statistical analysis was performed using R Program (R Foundation for Statistical Computing), Version 2.15.2 (2012).

## Results

Number of recordings (N) required to produce statistically significant results (*p* < 0.05) was 11, with measured effects on upper trapezius, biceps brachii and triceps brachii being of such magnitude that a minimum of only three measurements would be required to get statistically significant data.

The mean elbow extension passive ranges of movement (ROM) during the pre-manipulative stretch were verified to be consistent at mean 181.6° (SD 1.9°) for the right tested side and 183.1°(SD 2.1°) for the left tested side during the Standard position, and 182.9°(SD 2°) and 184.1°(SD 1.7°) during the Varied position, right and left side respectively. These are consistent with those measured by Günal et al. [31].

**Figure 3:**
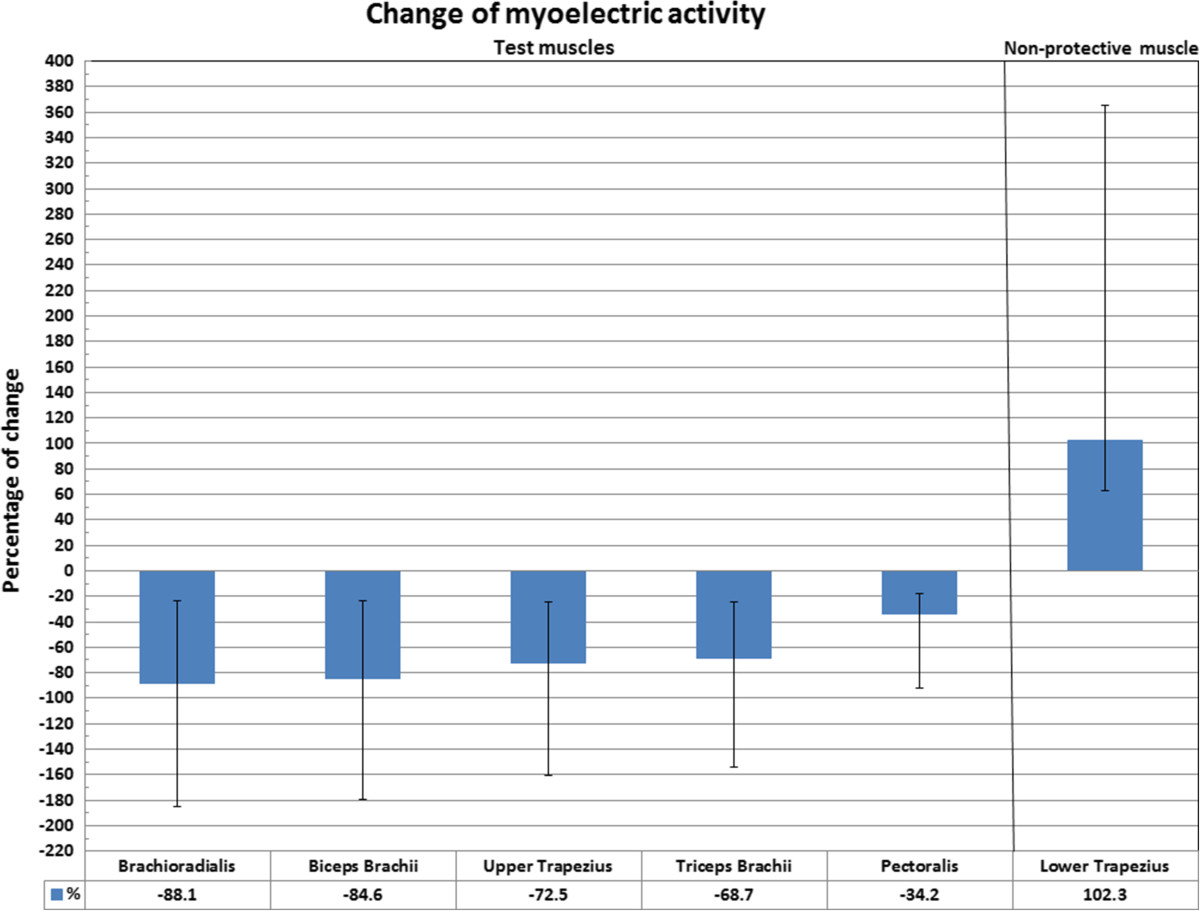
**Percent change in EMG activity of upper extremity muscles when the pre-manipulative stretch for Mills manipulation was performed in different shoulder positions (Standard versus Varied).** Confidence Intervals (CI) are presented.

**Figure 4:**
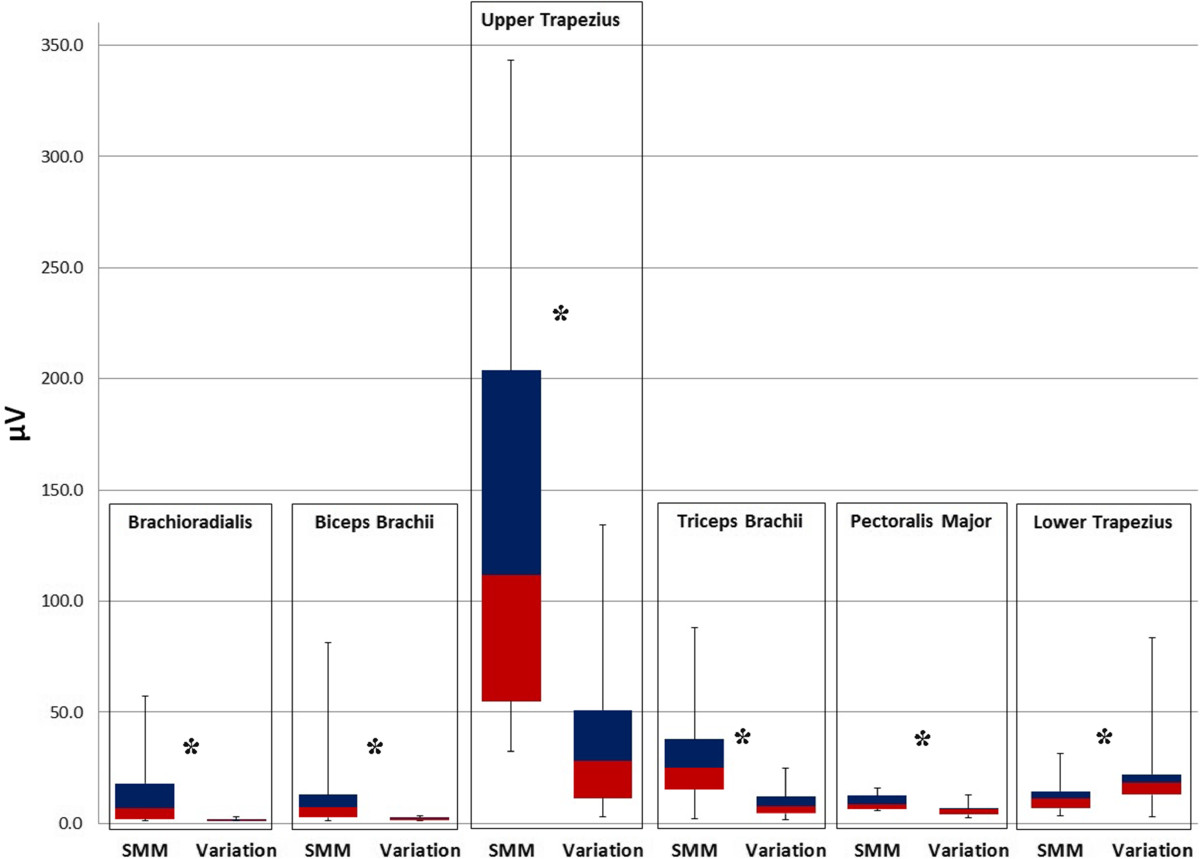
**Directional change in myoelectric values during the standard pre-manipulative stretch for Mills manipulation and the Varied position.** Significant changes (*P* < .05) are marked with an asterisk. Note the significant decrease of myoelectric activity and variance of the signal in all the test muscles in the Varied position. No significant difference in myoelectric activity was found between right and left sides (*P* > .05). Abbreviations: SMM - Standard Mills Manipulation.

The data showed that, compared with the Standard positioning, there was a significantly different pattern of muscle responses to the pre-manipulative stretch for Mills manipulation between the two positions of the shoulder. In the Varied position there was a significant reduction in myoelectric activity in all the test muscles, namely brachioradialis (*P* ≤ .001), biceps brachii (*P* ≤ .001), upper trapezius (*P* ≤ .001), triceps brachii (*P* ≤ .001), pectoralis major (*P* ≤ .001) and a significant increase of myoelectic activity for lower trapezius (*P* ≤ .002), which was considered the non-protective muscle (Table 2, Figures 3 and 4).

**Table 2:** **Myoelectric values expressed in micro volts (μV) for test and control muscles during Simulated Mills Manipulation**

		Standard Mills position	Varied position	Mean percentage change in myoelectric activity	*P* value	Observed power	Effect size
**Test muscles**	**Brachioradialis**	Mean 13.71 (SD 16.34)	Mean 1.63 (SD 0.61)	−88.1%	≤.001	0.99	0.73
	**Biceps Brachii**	Mean 14.44 (SD 21.2)	Mean 2.26 (SD 0.80)	−84.56%	≤.001	1	0.58
	**Upper Trapezius**	Mean 134.63 (SD 99.51)	Mean 37.08 (SD 33.58)	−72.46%	≤.001	1	1.31
	**Triceps Brachii**	Mean 28.71 (SD 20.67)	Mean 8.98 (SD 6.46)	−68.73%	≤.001	1	1.27
	**Pectoralis Major**	Mean 9.64 (SD 3)	Mean 6.34 (SD 2.92)	−34.18%	≤.001	0.99	1.14
**Non-protective muscle**	**Lower Trapezius**	Mean 12.22 (SD 7.26)	Mean 24.71 (SD 21.64)	+102.25%	≤.001	0.92	0.63

### Subjective data results

From the subjective questionnaire it emerged that, of the two positions in which the pre-manipulative stretch was performed, Standard (0° forward flexion) and Varied (65° forward flexion), 100% (16 out of 16) of the tested subjects reported the position of 65° forward flexion of the shoulder to be the less painful one.

## Discussion

It was shown that amplitude of myoelectric activity was significantly lower (*P* ≤ .001) in all the tested muscles during the pre-manipulative stretch in the Varied position compared with the Standard. The Varied position encompassing shoulder girdle forward flexion to 65° likely decreased the mechanical tension in the brachial plexus by i) decreasing the distance between the lower cervical region and the axilla, ii) increasing the area of the thoracic outlet tunnel as the clavicle moves anteriorly away from the first and second ribs, while not modifying any other component of the Mills manipulation manoeuvre (Figure 1).

While all the tested muscles showed a highly significant decrease in EMG activity, the largest change was in muscles that may exert a protective effect on the radial nerve and its posterior interosseus branch by means of shortening their pathway in front of the elbow joint and shoulder. These muscles consist of biceps brachii, brachioradialis and upper trapezius.

The results in this study are consistent with those of other investigations in which the shoulder girdle has been shown to elevate [28] and the upper trapezius muscle has been shown to participate in this effect [32] in response to the neurodynamic test for the brachial plexus and median nerve.

The neurodynamic test for the median nerve has been shown by Kleinrensink et al. [7] to apply tensile force to this nerve into the axilla, but also the medial and lateral cords of the brachial plexus by a similar amount as has been shown in the same study to also occur in the RNT. Based on this, shoulder girdle elevation may exert a protective effect in which the upper trapezius muscle activity produces a reduction in the distance between the lower cervical spine and the axilla, therefore reducing tension in the brachial plexus.

It could be hypothesized that certain muscle activity during increases in mechanical tension in the peripheral nerves is a nociceptive flexor mediated reflex. However, in Balster and Jull [32], the lack of correlation between the magnitude of painful symptoms at the end range elbow of extension and cervical contralateral lateral flexion stages (which increases tension in the nerves) during neurodynamic test for the median nerve and the collected EMG data on the ipsilateral upper trapezius, suggests that the mechanism may be more complex.

The non-protective muscle, lower trapezius, showed a significant increase in myoelectric activity in the position involving shoulder 65° forward flexion. This may be interpreted in relation to its function as a scapular stabilizer in shoulder girdle abduction [33] and its contribution to posterior tilt and external rotation of the scapula during humeral elevation in standing or seated subjects [34].

The opposite myoelectric trend seen with in lower trapezius suggests that the overall changes in myoelectric activity in the test muscles may have been an expression of a specific protective response related to mechanical force production in the neural tissues, and not just the effect of a general increase of myoelectric activity.

In order to support the hypothesis that muscles can be directly activated to protect peripheral nerves from tensile and compressive mechanical loads, it would be necessary to demonstrate the existence of nerve terminals in the connective tissues of peripheral nerve and ectopic electrogenesis at the peripheral nerve level, either from those nerve terminals or the axons passing along the nerve. In 1963 Hromada [35] described the innervation of the connective tissues of peripheral nerves by nerve fibers termed “nervi nervorum”, whilst Bove and Light [36] reported neurovascular bundle nociceptors (nervi vasa nervorum) that responded to mechanical stimulation, which indicates that the observed responses to mechanical stimulation were not exclusively linked to discharge due to injury and in fact could be within the natural physiological receptive capabilities of peripheral nerve. The presence of C nociceptive and compression/stretch-responsive endings in the neural connective tissue (eg. epineurium) supports the notion that ectopic electrogenesis could indeed be produced by mechanical forces applied to the nerve trunk, which helps explain the findings presented in this paper.

The myoelectric signals presented in this paper were recorded in different pre-manipulative positions to describe the protective reaction induced by neural stretching.

The recorded myoelectric signals in response to these passive movements showed considerably low amplitude if compared to those usually found in active movements. Even if proper steps were taken to detect and avoid power line interferences and background noise into the recorded signals, in future investigations it may be appropriate to also record myoelectric activity during rest in order to unequivocally exclude such interferences.

Moreover, the position of surface electrodes may change with respect to the muscle fibres from the underlying muscles, particularly during shoulder movements. This is a common issue with surface electromyography that may be corrected with the use of needle electrodes. The authors would like to list these points both as limitations of the present investigation and suggestions for further improvement.

In this study, the myoelectric reaction of asymptomatic subjects was assessed in order to provide normative data that will allow for future clinical comparisons in patients with epicondylalgia. Further investigations should be conducted in tennis elbow patients before generalizing the presented results to a symptomatic population.

## Conclusions

Based on the results presented in this study, it appeared that dual muscle and nerve mechanisms may have operated during CEO stretch, suggesting that Mills manipulation may have collateral effects that may need controlling during the clinical procedure.

If this paradigm can be applied to the clinically applied Mills manipulation, it seems that performance of the manoeuvre in 65° forward flexion of the shoulder girdle joint may reduce neural tension and extraneous muscle responses. This is on the basis of reductions in both myoelectric activity and reported symptoms by the subjects with the forward flexed position. An advantage of this notion is that the non-specific responses may be reduced while the key movements of the manipulation and the local, or ‘targeted’ effects, would remain unchanged.

Normative data that will allow for future clinical comparisons when evaluating this proposal have here been presented (Table 3).

**Table 3:** **List of conclusions**

Findings	Cautions
• It is here hypothesized that the radial nerve and its posterior interosseus branch are stretched during the execution of a Standard Mills manipulation, and that the muscles are being selectively activated in order to protect the peripheral nerves in the most logical way; by shortening the neural pathway and opposing the manipulation movement.	• Further investigations should be conducted in tennis elbow patients before generalizing the presented results to a symptomatic population.
• All the Test muscles showed a statistically significant decrease in myoelectric activity (*P* ≤ .001) in response to forward flexion of the shoulder to 65° during Mills manipulation (condition of reduced nerve tension), showing a correlation between likely neural tension and myoelectric activity in protective muscles.	• The currently hypothesized mechanisms linking muscle activation and the raise of mechanical tension in the peripheral nerves is the nociceptive flexor mediated reflex (NFR). However, more research may be needed to fully understand this phenomenon.

It appeared that the reduction in muscle activity in the position of 65°shoulder girdle forward flexion during the pre-manipulative stretch for Mills manipulation, compared with performance of the stretch in the standard position, suggested that a neural mechanism influenced the muscle responses. It seemed possible that the muscles were reflexively activated in order to protect the peripheral nerves in the most logical way; by shortening their pathway and opposing the manipulation movement. This is interesting in relation to the possibility that these results may later be compared with data collected from patients with lateral epicondylalgia. There also seems justification for further investigation in order to elucidate the relevant mechanisms.

## Authors’ original submitted files for images

Below are the links to the authors’ original submitted files for images. Authors’ original file for figure 1 Authors’ original file for figure 2 Authors’ original file for figure 3 Authors’ original file for figure 4

## Abbreviations

A/D board: Analogue to digital conversion board
CCD: Charge-coupled device
CEO: Common extensor origin
EMG: Electromyography
MVC: Maximal voluntary Contraction
RNT: Radial neurodynamic test
ROM: Range of movement
SMM: Standard Mills Manipulation.
