# Supplementary figures and images for: Effect of glenohumeral forward flexion on upper limb myoelectric activity during simulated mills manipulation; relations to peripheral nerve biomechanics
Source: BMC Musculoskelet Disord. 2014 Sep 2;15:288. doi: 10.1186/1471-2474-15-288 (PMC4161896; doi:10.1186/1471-2474-15-288)

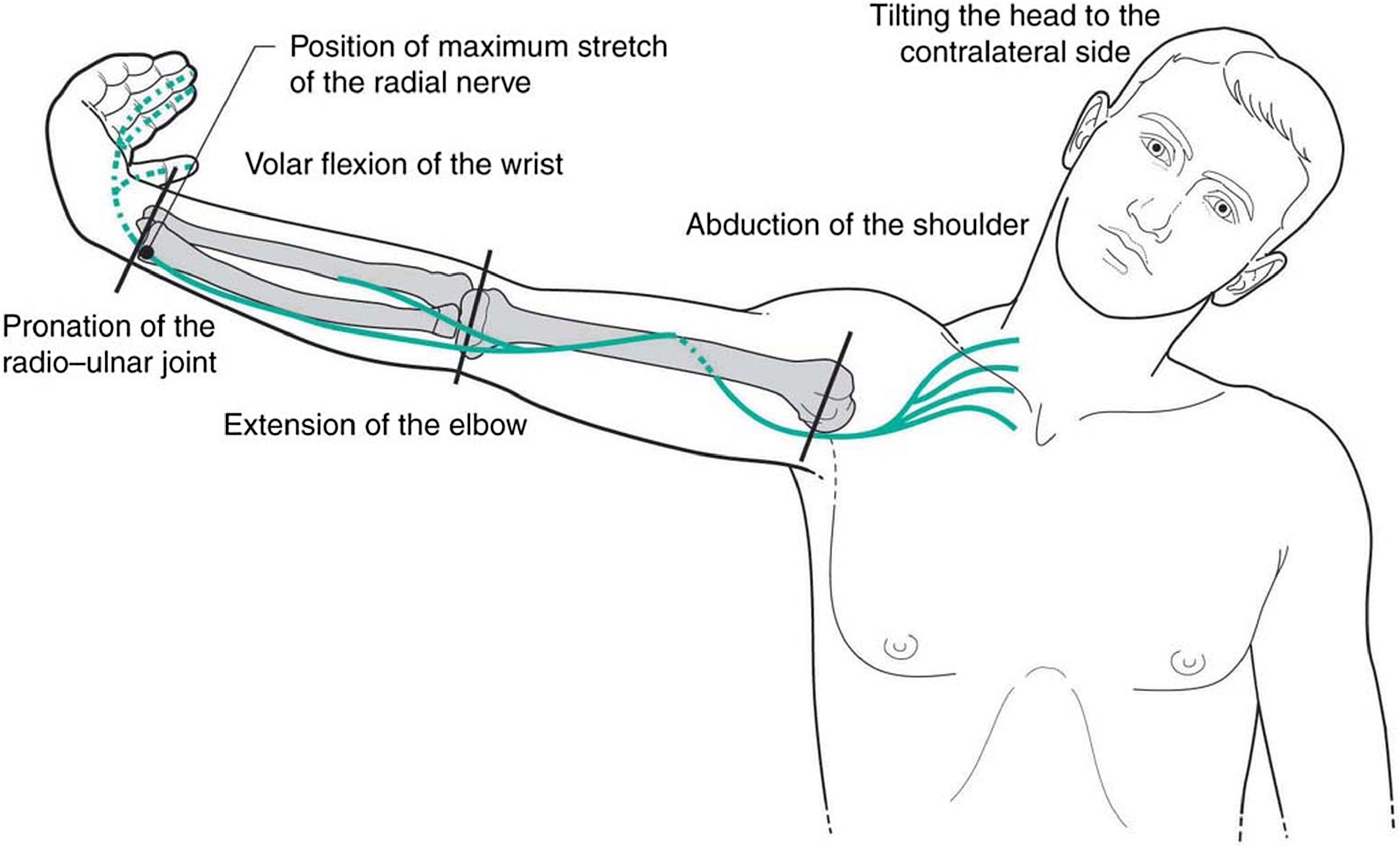

Supplement: Supplementary file 1 — Authors’ original file for figure 1 [file 12891_2014_2236_MOESM1_ESM.tif]

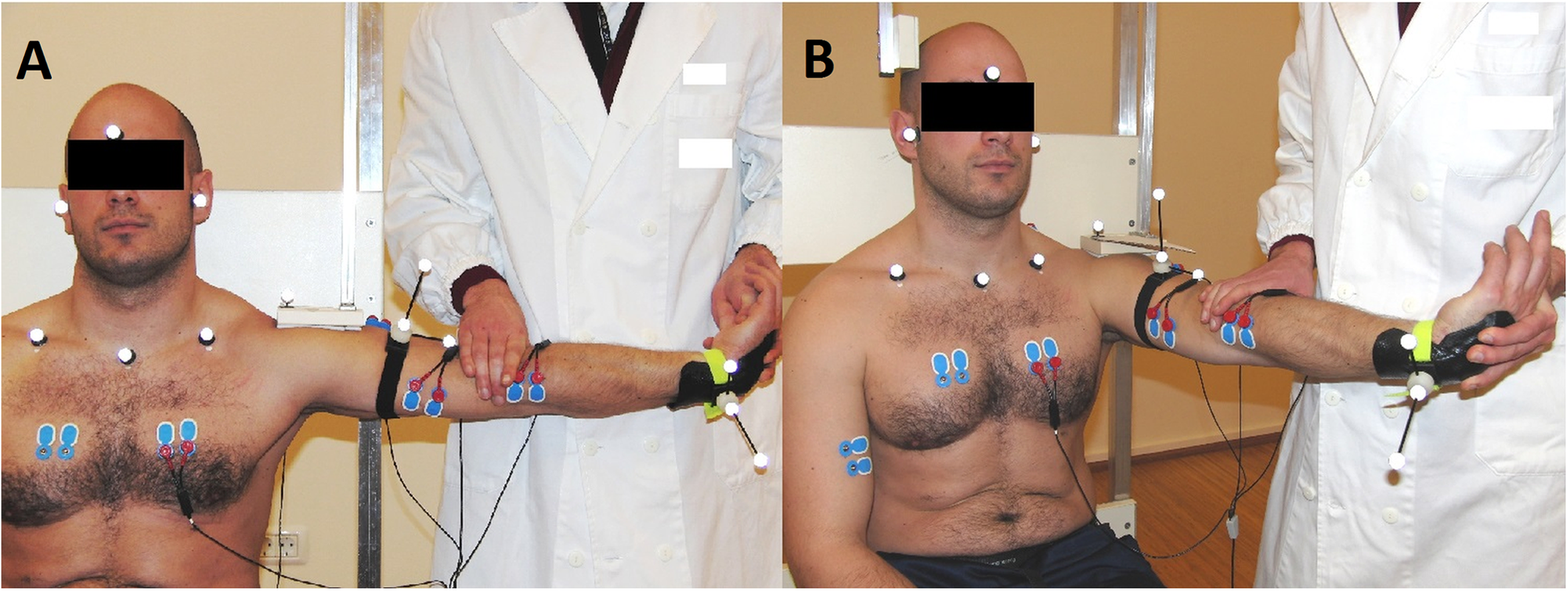

Supplement: Supplementary file 2 — Authors’ original file for figure 2 [file 12891_2014_2236_MOESM2_ESM.tiff]

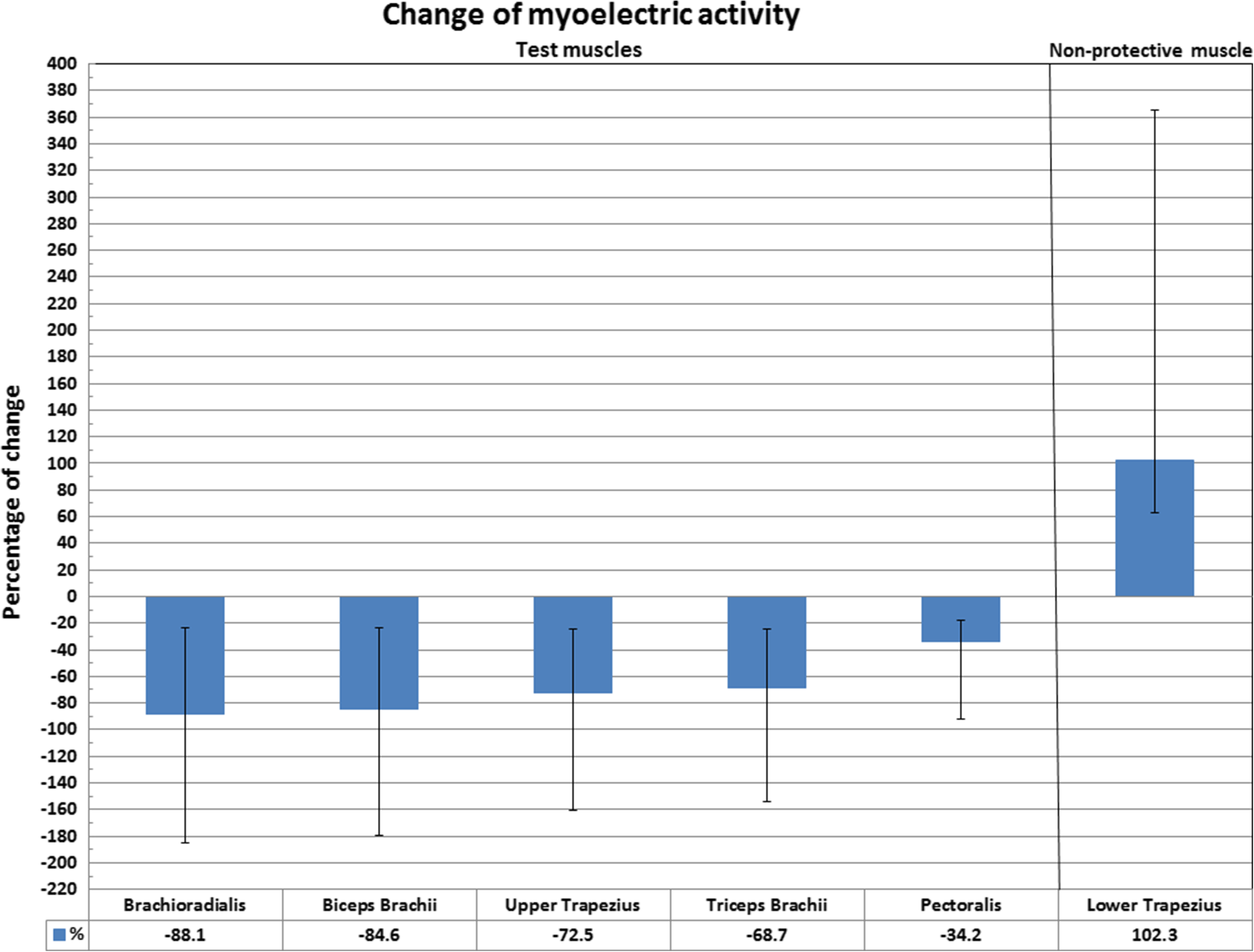

Supplement: Supplementary file 3 — Authors’ original file for figure 3 [file 12891_2014_2236_MOESM3_ESM.tif]

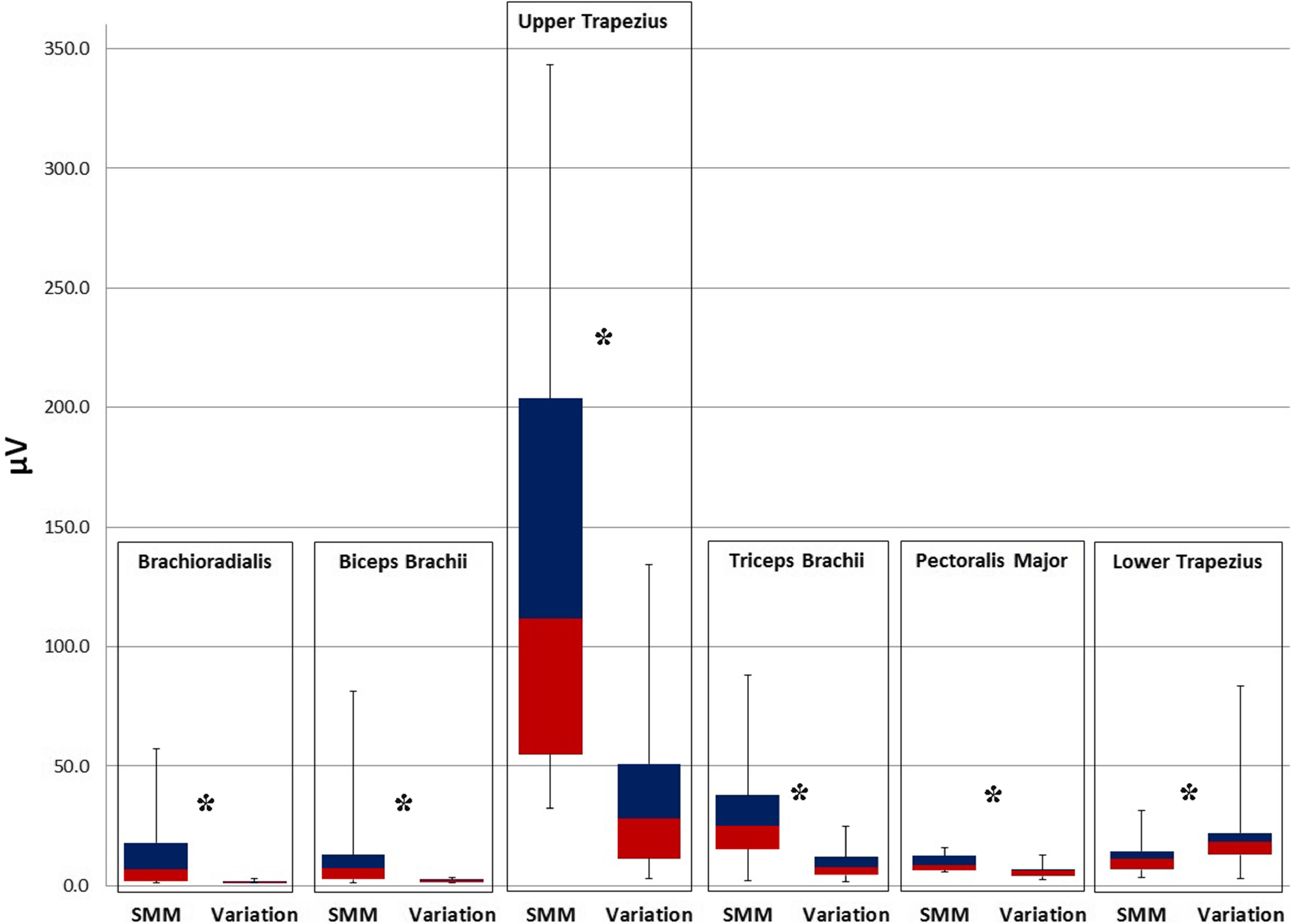

Supplement: Supplementary file 4 — Authors’ original file for figure 4 [file 12891_2014_2236_MOESM4_ESM.tif]
